# A Treatise on Materia Medica, Pharmacology and Therapeutics

**Published:** 1890-03

**Authors:** 


**A Treatise on Materia Medica, Pharmacology, and Therapeutics.** By JOHN V. SHOEMAKER, A. M., M. D., and JOHN AULDE, M. D., Philadelphia and London. F. A. Davis, Publisher. 1889. In two volumes. Vol. I., 353 pages, with sixty-six blank pages for notes. Price, cloth, $2.50 ; sheep, $3.25.

A new work on therapeutics must cut loose from the traditional heresies that have been handed down from time immemorial, but it should not condemn as absolute, empirical methods which have shown their value in times gone by; on the contrary, we should strive to learn the secret of the successful application of remedies that baffled the generation of physicians before us.

Such a determination on the part of the authors in the preface of their work, naturally causes the reader to expect something out of the ordinary run of text-books. Perhaps, in no branch of medicine can there be as much originality shown in treating and discussing a subject as in materia medica and therapeutics. A new text-book on therapeutics, in order to be successful, must, therefore, present its matter in an original and convincing manner.

The authors seem to have been cognizant of this fact, and have stamped it on every page of Vol. I. We await with pleasure the appearance of Vol. II., and hope to find in it a work equally as interesting and valuable as Vol. I.

Part I. is devoted to Materia Medica, Pharmacy, Pharmacology and Therapeutics. Under the head of Materia Medica is placed in tabulated lists the natural distribution of the remedies at our command, comprising inorganic and organic materia medica. Pharmacy, as the true art in medicine, is considered of such importance to the physician that the authors have described the more important pharmacopeial processes and preparations in use in the laboratory and dispensary. This part of the work give the physician and student an insight into the technique of compounding and dispensing medicines, and cannot but prove beneficial.

The classification of medicine is largely that of Garrod, modified to some extent by the teachings of Brunton. The three general classes, medicines for internal administration, external applications, and chemical agents as antidotes, disinfectants, and antiseptics, are all carefully and methodically analyzed, accompanied by diagrams showing the comparative value of the different drugs and mode of action. Perhaps in no subject is the saying, "classification is everything," so cogent as in pharmacology. The authors have rendered this work of inestimable value through the many carefully compiled tables which it contains, facilitating the study of drugs as no other method can do.

Part II. treats of remedies and remedial agents used in the treatment of diseases not properly classed as drugs. The chapter on electro-therapeutics embraces a thorough study of the constant, interrupted, and static electricity with their indications and mode of administration. Then follow chapters on Oxygen, Hydro-therapeutics, Masso-therapeutics, Heat and Cold, Mineral Waters, Metallo-therapy, Transfusion, Hypnotism and Suggestion, Earth Dressing, Baunscheidtismus, Climatology, Light, Music, Blood Letting, and Suspension, making a complete encyclopedia of therapeutical knowledge. Some of these agents, now of recognized therapeutical worth, having been slighted by many of the older works, receive in this book full and frank consideration.

Paper, print, and binding help to make the book redound with praise to editors and publishers alike. W. C. K.

